# Renal Function and NODM in *De Novo* Renal Transplant Recipients Treated with Standard and Reduced Levels of Tacrolimus in Combination with EC-MPS

**DOI:** 10.1155/2012/941640

**Published:** 2012-11-25

**Authors:** Laurence Chan, Amado Andres, Suphamai Bunnapradist, Kristene Gugliuzza, Ravi Parasuraman, V. Ram Peddi, Elisabeth Cassuto, Marquis Hart

**Affiliations:** ^1^Transplant Center, University of Colorado Denver, Aurora, CO 80045, USA; ^2^Servicio de Nefrologia, Hospital Universitario 12 de Octubre, 28041 Madrid, Spain; ^3^UCLA David Geffen School of Medicine, University of California, Los Angeles, CA 90048, USA; ^4^Medical Branch, University of Texas, Galveston, TX 77555, USA; ^5^Nephrology and Hypertension, Henry Ford Hospital, Detroit, MI 48202-2689, USA; ^6^California Pacific Medical Center, San Francisco, CA 94115, USA; ^7^Néphrologie, Dialyse et Transplantation, Hôpital Pasteur, 06002 Nice, France; ^8^UCSD Medical Center, San Diego, CA 92103-8401, USA

## Abstract

Information is lacking concerning concomitant administration of enteric-coated mycophenolate sodium with tacrolimus (EC-MPS+Tac) in renal transplant recipients (RTxR). In this 6-month, prospective, open-label, multicenter study, *de novo* RTxR were randomized (1 : 1) to low-dose (LD) or standard-dose (SD) Tac with basiliximab, EC-MPS 720 mg bid, and steroids. Primary objective was to compare renal function at 6-month posttransplantation. Secondary objectives were to compare the incidences of biopsy-proven acute rejection (BPAR), graft loss and death, and new-onset diabetes mellitus (NODM). 292 patients (LD *n* = 151, SD *n* = 141) were included. Mean Tac levels were at the low end of the target range in standard-exposure patients (SD, *n* = 141) and exceeded target range in low-exposure patients (LD = 151) throughout the study. There was no significant difference in mean glomerular filtration rate (GFR) between treatments (ITT-population: 63.6 versus 61.0 mL/min). Incidence of BPAR was similar (10.6% versus 9.9%). NODM was significantly less frequent in LD Tac (17% versus 31%; *P* = 0.02); other adverse effects (AEs) were comparable. EC-MPS+Tac (LD/SD) was efficacious and well tolerated with well-preserved renal function. No renal function benefits were demonstrated, possibly related to poor adherence to reduced Tac exposure.

## 1. Introduction

Mycophenolate mofetil (MMF), a prodrug of mycophenolic acid (MPA), is associated with a high incidence of gastrointestinal (GI) AEs [1, 2]. Enteric-coated mycophenolate sodium (EC-MPS) is an MPA formulation developed to improve MPA-related upper GI side effects by delaying the release of MPA until reaching the small intestine. Clinical trials in renal transplant (RTx) patients have demonstrated therapeutic equivalence between EC-MPS and MMF when administered at equimolar dosages [3–5], and data suggests a reduced GI-related symptom burden and improved patient well-being with EC-MPS treatment compared with MMF [6–8]. However, Phase III trials with both MMF and EC-MPS were all performed in patients on a cyclosporine (CsA) microemulsion-based immunosuppressive regimen; there is limited experience with the use of EC-MPS in combination with tacrolimus (Tac). It has been shown that conversion of Tac-treated maintenance RTx patients from MMF to EC-MPS was well tolerated without compromising efficacy [9], but more rigorous data is required on efficacy and safety of EC-MPS with Tac. 

The use of Tac/MMF combination has increased in recent years and it is now widely used in transplantation [10–12]. With regard to pharmacokinetics, in contrast to Tac, CsA interrupts the enterohepatic recirculation of MPA by inhibition of the multidrug resistance protein (Mrp)-2, which excretes MPA 7-O-glucuronide (MPAG) into bile [13, 14]. Consequently, the area under the plasma concentration-time curve (AUC) for MPA in patients receiving MMF/Tac is about 35% higher than in patients receiving MMF/CsA [15]. This has led to recommendations that MMF dose be adjusted in patients receiving Tac [15, 16]. In fact, MMF doses during Tac comedication are around 30% to 50% lower than those used with CsA [17]. With EC-MPS, a modest degree of change in MPA exposure has been reported in a randomized calcineurin inhibitor (CNI) crossover study in stable RTx patients; MPA AUC was 19% higher during concomitant treatment with Tac versus CsA [18]. 

Recently, several studies investigated low-dose Tac/MMF regimens in RTx recipients [19–21]. These studies indicated that a regimen of continuous low-dose Tac/MMF may reduce Tac-related AEs without compromising efficacy. With EC-MPS, no such study has generated data up to this point. Here we summarize the results of a study designed to investigate the safety and efficacy of EC-MPS with both standard and reduced Tac levels in *de novo* renal allograft recipients. 

## 2. Materials and Methods

### 2.1. Study Design and Patient Population

This was a 6-month, prospective, randomized, open-label, parallel-group study conducted at 32 centers in 7 countries (Canada, France, Italy, Poland, Spain, UK, and USA) in patients (18–70 years) of low immunologic risk (defined as patients with panel reactive antibodies (PRA) <20%, no retransplantation, no ABO incompatible, and cold ischemia time (CIT) <30 h) who had received their first renal allograft. Recipients of human leukocyte antigen (HLA)-identical living-related allograft were excluded. Other main exclusion criteria comprised: multiorgan transplant, donation after cardiac death, females of child-bearing potential, donor age >65 years, CIT >30 h, panel-reactive antibodies of >20% prior to transplantation, and positive test for hepatitis B or C virus of donor or recipient. Approval was obtained from the Institutional Review Committee of each participating center, and written informed consent was received from each patient prior to recruitment. The trial was performed in accordance with the amended Declaration of Helsinki.

Patients were randomized (1 : 1) to one of two treatment regimens, within 24 h after transplantation.

1. Group A (low-dose Tac group): basiliximab induction, EC-MPS, cortico-steroids (CS), and low-dose Tac (target trough level of 5–9 ng/mL for the first 3 months and 3–6 ng/mL for the subsequent 3 months).
2. Group B (standard-dose Tac group): basiliximab induction, EC-MPS, CS, and standard-dose Tac (target trough level of 10–15 ng/mL for the first 3 months and 8–12 ng/mL for the subsequent 3 months).

In both groups, the EC-MPS (Myfortic, Novartis Pharma AG, Basel, Switzerland) dose was 1440 mg/day (720 mg twice daily, equimolar to MMF 2 g/day). Dose adjustments were permitted at the discretion of the investigator. EC-MPS and Tac (Prograf, Astellas Pharma, Tokyo, Japan) were initiated within 24 h after reperfusion of the graft. If patients experienced delayed graft function, Tac could be withheld for up to 7 days. Initial doses of Tac were selected as per local practice to be adjusted according to the protocol defined target trough levels. The dosing regimen of basiliximab (Simulect, Novartis Pharma AG, Basel, Switzerland) consisted of two intravenous doses of 20 mg each, administered within 2 h before, and 4 days after, transplant surgery. All patients received intravenous methylprednisolone pre- or intraoperatively. Treatment with oral prednisone (or equivalent) was initiated on day 1 posttransplantation at a minimum daily dose of 20 mg (tapered as per center practice to a minimum maintenance dose of 5 mg/day). The randomization list was generated by Novartis Pharma AG using a validated system that automates the random assignment of treatment arms to randomization numbers in the specified ratio. All patients were followed until 6 months after transplantation, unless they withdrew informed consent, died, or were lost to followup.

### 2.2. Study Endpoints

Primary efficacy endpoint was renal function at 6-month posttransplant, as assessed by estimated GFR using the Nankivell formula [22]. Key secondary efficacy endpoints included treatment failure rate (a composite of BPAR, graft loss, or death) and its individual components at month 6, serum creatinine and calculated 6-month creatinine clearance (CrCl) using the Cockcroft-Gault formula [23]. In addition to the incidences of AEs (including infections) and serious adverse events (SAEs), safety endpoints included the incidence of NODM within the first 6 months of transplantation among patients classified as nondiabetic at time of transplant surgery. Diagnosis of NODM was based on criteria specified by the American Diabetes Association (ADA/WHO) [24]. Treated diabetes was defined as the use of any oral hypoglycemic medication or insulin for a minimum period of 14 consecutive days during the study period.

### 2.3. Procedures

 In general, evaluations were performed at baseline, days 2, 4, 6, weeks 2 and 4, and at months 3 and 6 or at premature discontinuation. Laboratory samples (including serum creatinine and fasting plasma glucose) were analyzed centrally. Tac whole blood levels were measured at local laboratories. An oral glucose tolerance test (OGTT) following WHO guidelines [25] was performed after 3 and 6-month posttransplant and analyzed locally. Unless medically contraindicated, all suspected rejection episodes required confirmation by core renal biopsy to be read locally. BPAR was classified according to the Banff criteria [26]. All AEs and SAEs were monitored throughout the study. 

### 2.4. Statistical Methods

Primary efficacy variable, estimated GFR, was used for the sample size calculation. Sample size estimation of 288 patients (144 per treatment group) was calculated, assuming a dropout rate of 3% for death and graft loss. For the latter patients, GFR was to be imputed as 0; for other missing values, the last observation carried forward (LOCF) procedure was to be applied. The planned number of patients would provide a power of 80% to detect a difference of at least 7 mL/min/1.73 m^2^ in mean GFR at a two-sided significance level of 0.05, assuming a GFR in the control group (Group B) of 50 mL/min/1.73 m^2^ with a standard deviation of 20 mL/min/1.73 m^2^. Analysis of variance (ANOVA) was used to compare continuous endpoints (GFR, CrCl, serum creatinine) including treatment and country in the model. Least squares means and associated two-sided 95% confidence intervals (CIs) for the differences are presented. Categorical variables were tested using Fisher's exact test or the chi-square test as appropriate. Treatment failure and its individual components were analyzed using Kaplan-Meier (KM) estimates and the Logrank test. For all statistical tests, *P* value < 0.05 was considered significant.

Efficacy analyses were performed on the intent-to-treat (ITT) population, which comprised all randomized patients. Patient population evaluated for safety included all randomized patients who received at least one dose of EC-MPS and had at least one safety evaluation. In this study, the ITT and safety populations were identical. In addition, an observed cases analysis of GFR data was carried out on the ITT population without imputation for missing data.

Because of unsatisfactory adherence to the protocol defined Tac trough levels in the present study, an additional analysis (data-driven analysis) was carried out based on the data of those ITT patients who showed a consistent pattern of adherence to one of the two Tac ranges (regardless of patient randomization). These patients were defined “as per Tac target level population.” This post hoc analysis was driven by the high proportion (67.5%) of randomized patients with ≥50% of their Tac trough levels outside the target ranges. For this analysis, all Tac trough concentrations collected during the study were analyzed in a blinded fashion by three independent reviewers. Consistent adherence of a patient was defined as >50% of Tac levels (mandatory for the month 6 trough level) being within the Tac target range defined in the study protocol. Within the “as per Tac level population,” patients were re-classified to one of two groups: either “reclassified” Group A or “reclassified” Group B. The post-hoc analysis compared 6-month GFR data between these groups; the statistical methods applied were the same as defined for the primary analysis.

## 3. Results

### 3.1. Patient Disposition and Baseline Characteristics

A total of 303 patients were screened, out of which 292 were randomized: Group A, 151; Group B, 141. More than 80% of patients in each group completed study treatment with no discernable difference in terms of discontinuation rate. The main reason for patients discontinuing study drug was AE experience (Group A, 7.9%; Group B, 10.6%). The percentage of patients who did not complete 6-month followup was comparable between groups (Group A, 6.6%; Group B, 4.3%), with withdrawal of consent and lost to followup being the most common reasons.

Overall demographic and baseline characteristics were comparable between groups, except for a larger percentage of black patients in Group B (4.6% versus 10.6%) (Table 1). Delayed graft function was reported for 24.3% of patients (Group A, 24.5%; Group B, 24.1%). 

### 3.2. Immunosuppressant Dose and Exposure

The mean daily dose of EC-MPS over the entire study period was similar across treatment groups (Group A, 1296 ± 225 mg; Group B, 1325 ± 206 mg) as was the cumulative steroid dose (Group A, 3033 ± 1623 mg; Group B, 3020 ± 1414 mg). 

Four patients (3 randomized to Group A and 1 to Group B) did not receive Tac. Two patients (in Group A) did not report the use of basiliximab. 

**Figure 1:**
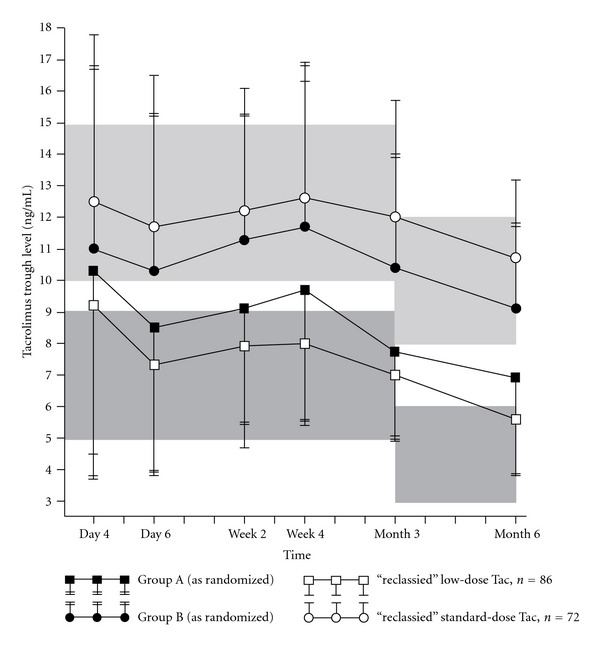
Mean tacrolimus trough levels over time. Tac = tacrolimus; Group A = low-dose tacrolimus (*n* = 151); Group B = standard-dose tacrolimus (*n* = 141); “reclassified” defined as >50% of Tac levels (mandatory requirement for month 6 assessment) being within a protocol-specified Tac target range. Bars represent one standard deviation. Shaded areas represent protocol specified target ranges for tacrolimus.

The average daily dose of Tac in Group A and B was 0.10 and 0.13 mg/kg (up to month 2), 0.08 and 0.12 mg/kg (month 3), and 0.08 mg/kg and 0.11 mg/kg (months 4 to 6), respectively. There was a marked trend towards the lower limit of the target range in the standard-dose group and towards the upper level limit in the reduced-dose group (Figure 1). Overall, two-thirds of Tac trough level measurements were outside the target ranges. In fact, patients in Group A were frequently above the Tac target range (24.4%–52.5% of patients across visits), while patients of Group B were commonly below target (31.5% to 53.3% across visits). As a consequence, the difference between the mean trough levels in the two groups was smaller than specified by the protocol. At all assessments, the treatment group difference in mean Tac trough concentrations was less than the 50% difference of the two target range midpoints (i.e., 5.5 ng/mL) established in the protocol. No reasons could be identified for the frequent nonadherence to prespecified Tac target ranges. Mean Tac trough levels of the reclassified “as per target Tac level population” (*n* = 158), (i.e., patients with a sufficient adherence to Tac target ranges) are displayed in Figure 1.

## 4. Efficacy

### 4.1. Renal Function

 At month 6, there was no significant difference in mean GFR between treatment groups (Table 2). Likewise, there was no difference in mean creatinine clearance (CrCl) (Group A, 62.1 mL/min; Group B, 59.5 mL/min, *P* = 0.411) and mean serum creatinine (Group A, 144.0 *μ*mol/L; Group B, 135.3 *μ*mol/L, *P* = 0.373). In the observed cases analysis (no values imputed or carried forward), a statistical trend was observed towards an improvement of kidney function in patients receiving a reduced Tac dose regimen (Table 2). Analysis on the “as per target Tac level” population showed a significantly better renal function (almost reaching the stipulated GFR difference) in patients with lower Tac trough concentrations, with a difference of 6.6 mL/min/1.73 m^2^ in GFR between “reclassified” Group A and “reclassified” Group B (Table 2). 

### 4.2. Biopsy-Proven Acute Rejection (BPAR) and Treatment Outcomes

After 6 months, no difference was observed between Group A and Group B with respect to treatment failure KM estimates: 0.034 (95% CI −0.044–0.112); *P* = 0.397) or BPAR alone (KM estimates: 0.010 (95% CI −0.062–0.081); *P* = 0.804) (see Table 1: Supplemental digital content in supplementary material available online at doi:10.1155/2012/941640). Three graft losses of Group A were considered unrelated to the Tac dosing regimen; two occurred at the first day of EC-MPS dosing without concomitant Tac and one hyper acute humoral rejection was reported on day 3. Other reasons for graft loss were infarcted kidney (2), disease recurrence, primary graft nonfunction, and renal artery stenosis. Deaths reported (Table 3) were due to septic shock (Group A), and cardiac arrest and intra-abdominal hemorrhage (Group B). Treatment group differences in graft loss and death were not statistically significant. In “as per target Tac level population,” there was no statistical difference in treatment failure between “reclassified” Group A and “re-classified Group B (11 versus 10 patients). Similarly, no statistical difference was observed with BPAR alone (BPAR: 2 versus 0). 

## 5. Safety

The proportion of patients who experienced at least one AE or serious adverse events (SAE) was similar between treatment groups (Table 3). The most commonly reported AEs were infections, GI-events, and anemia, without a discernable difference between the two groups. In particular, there was no clinically relevant difference in the incidence and severity of cytomegalovirus infections or bacterial infections. Tremor was more frequently reported in Group B (*P* = 0.045). The most commonly reported SAE was increased creatinine (approximately 8% of patients in both groups). Serious GI AEs were reported by 10.6% and 8.5% of patients in Groups A and B, respectively. Only 4 patients of each group discontinued EC-MPS due to GI AEs. Three malignant neoplasms occurred during the study: renal cell carcinoma (Group A); basal cell carcinoma and malignant melanoma (both in Group B). In “as per Tac target level population, the proportion of patients experiencing at least one AE were similar between groups (97.7% versus 98.6%). The most commonly occurring (≥10%) AEs reported in “re-classified” Group A and “re-classified Group B were diarrhea (39.5 versus 37.5), constipation (43.0 versus 29.2), nausea (27.9 versus 31.9), and vomiting (16.3 versus 15.3).

**Figure 2:**
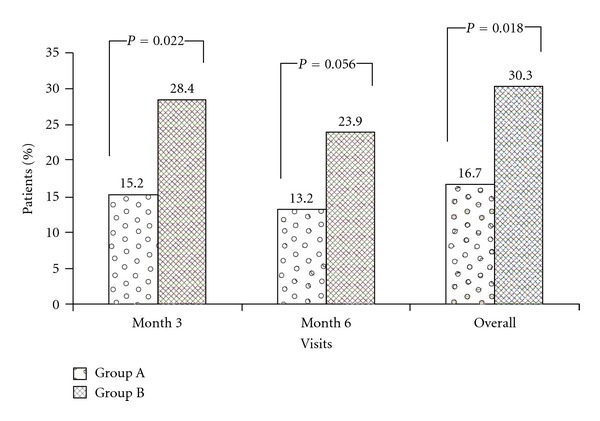
Incidence of new onset diabetes mellitus at month 3, 6, and overall (safety population Group A, *n* = 114; Group B, *n* = 109). NODM = patients treated for hyperglycemia for a period of 14 consecutive days, or had 2 h OGTT posttest value ≥200 mg/dL, or had at least two fasting glucose values ≥126 mg/dL, or had one single random value (fasting or nonfasting) ≥mg/dL. Safety population, patients with diabetes mellitus active at start of study, was excluded.

New-onset diabetes mellitus occurred in 19/114 patients of Group A (16.7%) and 33/109 patients of Group B (30.3%, *P* = 0.018) (Figure 2). The incidence of treated diabetes alone was also lower in Group A than in Group B (10.5% versus 17.4%) but the difference did not reach statistical significance. The incidence of new onset of insulin dependence was 4.4% in Group A and 8.3% in Group B (*P* = 0.277). No statistically significant or clinically relevant differences between the two treatment groups were seen for mean hematology and biochemistry parameters. 

## 6. Discussion

This is the first study which prospectively assessed the safety and efficacy of EC-MPS in combination with Tac (standard and reduced dose) in *de novo* renal allograft recipients. There is an increasing interest in immunosuppression strategies that allow reduction or elimination of CNIs with the aim to reduce associated toxicities, particularly nephrotoxicity [27]. 

This study demonstrated that in the first 6 months posttransplant, the combination of EC-MPS/Tac (at standard and reduced levels) is efficacious and well tolerated in *de novo* RTx recipients, confirming initial results on this combination assessed in the maintenance RTx population [9]. At 6 months, the incidences of treatment failure and BPAR were similar in both treatment groups and comparable to published reports using a similar immunosuppressive therapy [21, 28]. In particular, the efficacy results were comparable with a MMF/reduced-dose Tac regimen (BPAR, 11.3%) in a similar and large group of low-to-moderate risk patients when combined with anti-interleukin-2 receptor antibody induction [21]. The consistency of efficacy data with MMF studies confirms that equimolar MMF and EC-MPS doses are associated with similar MPA exposure and equivalent pharmacodynamic effects (inhibition of inosine 5′-monophosphate dehydrogenase activity) in patients on a Tac-based regimen [9]. 

With both Tac regimens, the GFR data in this study seems to be comparable with the results documented in the MMF-Tac arm of the trial performed by Ekberg and colleagues [21]. Although this study demonstrated a higher mean GFR in the lower Tac versus standard Tac arm, this difference was not statistically or clinically significant. This lack of significance may be due to the fact that, overall, two-thirds of Tac trough levels achieved in this study were outside the target ranges resulting in a smaller difference between the mean trough levels in the two treatment groups. Suboptimal adherence to protocol-defined target ranges during immunosuppressive therapy has been previously reported and complicates the interpretation of study results [29, 30]. A post hoc analysis of patients who consistently adhered to prespecified Tac ranges was performed in this study. In patients, who adhered to target ranges, mean GFR was significantly higher with lower Tac levels compared to those exposed to standard Tac concentrations. The resulting mean GFR with low-dose Tac (69.9 mL/min/1.73 m^2^) was comparable with other 6-month GFR data (using Nankivell or Cockcroft-Gault formulae) from studies investigating the possibility to use low-dose Tac regimens in RTx recipients [20, 31]. In the latter studies, no compliance problems with target ranges were reported.

Except for NODM, the type of AEs observed during treatment with EC-MPS and Tac were generally compatible with those observed in the *de novo* RTx population receiving EC-MPS/CsA [4]. Infections and GI complaints were the most frequently reported AEs in both groups. Moreover, AEs or SAEs reported were comparable with those reported with MMF/Tac regimens during the first 6 months posttransplant [28, 32]. In particular, there was no overt risk of cytomegalovirus (CMV) infections. The general absence of an expected lower incidence of AEs in the Group A of the current study may be due to the fact that there was a suboptimal adherence to protocol-defined Tac target ranges. 

The incidence of NODM was significantly higher with the standard-dose Tac regimen. The proportion of patients who developed NODM during standard-dose treatment was comparable with the Tac arm of a recent 6-month study which used a similar definition for NODM [28]. It has been reported that patients with high trough levels of Tac early after transplantation are prone to develop glycemic disorders and that the diabetogenic effect of Tac can be ameliorated by reducing the dose of Tac [33–35]. 

A limitation of the present study is the use of an open-label design with the potential for bias due to investigators' perceptions. Finally, followup was relatively short, although the majority of key events assessed (e.g., BPAR, NODM) are known to develop within the first 6 months posttransplant.

## 7. Conclusion

In conclusion, the results of our study show that EC-MPS in combination with standard and reduced Tac levels is effective and well tolerated in *de novo* RTx patients. Moreover, the safety and efficacy profiles of the EC-MPS/Tac regimens in this trial are comparable to that reported with MMF/Tac. With reduced Tac levels, the incidence of NODM is lower than with standard Tac concentrations without significant difference in short-term efficacy outcomes. In a subpopulation in which Tac target levels are met, significantly better renal function is observed with reduced Tac doses. 

## Supplementary Material

Supplementary Material shows additional information regarding efficacy failure rates (number of BPARs, worst severity of BPAR, treatment failure, and chronic nephropathy between the treatment groups in the ITT population)

## Figures and Tables

**Table 1:** Demographic and baseline characteristics between the treatment groups (ITT population).

	Low-dose Tac group (Group A) (*n* = 151)	Standard-dose Tac group (Group B) (*n* = 141)
Age (years)	47.7 ± 12.6	45.3 ± 12.9
Men (%)	72.2	65.2
Race (%)		
Caucasians	88.1	83.0
Blacks	4.6	10.6
Asians	4.6	3.5
Others	2.7	2.9
Time on dialysis (months)	30.6 ± 28.4	30.4 ± 27.1
Donor/recipient CMV serological status (%)		
Negative/negative	17.9	16.3
Negative/positive	19.9	20.6
Positive/negative	13.2	12.8
Positive/positive	43.7	48.2
Donor age (years)	42.7 ± 14.1	42.0 ± 13.9
Donor type (%)		
Donation after brain death	69.5	67.4
Living related	18.5	23.4
Living unrelated	11.9	9.2
Number of HLA mismatches (%)		
0	2.0	2.8
1–3	44.4	46.8
4–6	53.6	50.4
Cold ischemia time (h)	13.6 ± 9.1	12.0 ± 8.9
PRA < 20% (%)	97.4	98.6

CMV: cytomegalovirus; HLA: human leukocyte antigen, PRA: panel reactive antibodies.

Results expressed as mean ± standard deviation (SD) unless otherwise indicated.

**Table 2:** Renal function at month 6 assessed by estimated GFR (mL/min/1.73 m^2^) according to Nankivell formula.

Population	Low-dose Tac group (Group A)	Standard-dose Tac group (Group B)	Treatment difference (A−B)
ITT population	*n* = 145	*n* = 137	
Mean GFR	63.6	61.0	2.6
95% CI	58.8–68.4	56.2–65.9	−2.6–7.8
*P* value^1^			0.326

Observed-Cases^2^	*n* = 117	*n* = 111	
Mean GFR	69.5	65.4	4.2
95% CI	65.1–73.9	61.0–69.7	−0.5–8.8
*P* value^1^			0.079

“As per Tac level”	*n* = 85^3^	*n* = 69^3^	
Mean GFR	69.9	63.2	6.6
95% CI	63.1–76.6	56.0–70.4	0.4–12.9
*P* value^1^			0.038

CI: confidence interval, GFR: glomerular filtration rate.

^1^For A versus B (two-sided).

^2^ITT population without imputation for missing data.

^3^“reclassified” Group: defined as >50% of Tac levels (mandatory requirement for Month 6 assessment) being within the protocol-specified Tac target range.

**Table 3:** Adverse events (AEs) occurring in ≥20% of patients in any group, or those AEs of particular interest.

Events	Low-dose Tac group (Group A), (*N* = 151) *n* (%)	Standard-dose Tac group (Group B), (*N* = 141) *n* (%)
Any serious adverse event	73 (48.3)	67 (47.5)
Any infection	90 (59.6)	89 (63.1)
Bacterial	59 (39.1)	65 (46.1)
Viral	33 (21.9)	27 (19.1)
Any adverse event	145 (96.0)	138 (97.9)
Diarrhea	61 (40.4)	61 (43.3)
Nausea	47 (31.1)	47 (33.3)
Constipation	47 (31.1)	46 (32.6)
Urinary tract infection	43 (28.5)	44 (31.2)
Anemia	38 (25.2)	46 (32.6)
Procedural pain	31 (20.5)	33 (23.4)
Edema peripheral	34 (22.5)	25 (17.7)
Insomnia	21 (13.9)	32 (22.7)
Tremor	18 (11.9)	29 (20.6)^1^

^1^ *P* = 0.045 compared to Group A.
