# Supplementary material for: Renal Function and NODM in De Novo Renal Transplant Recipients Treated with Standard and Reduced Levels of Tacrolimus in Combination with EC-MPS
Source: J Transplant. 2012 Nov 25;2012:941640. doi: 10.1155/2012/941640 (PMC3512323; doi:10.1155/2012/941640)
Supplement: Supplementary file 1 — Supplementary Material shows additional information regarding efficacy failure rates (number of BPARs, worst severity of BPAR, treatment failure, and chronic nephropathy between the treatment groups in the ITT population) [file 941640.f1.docx]

**Supplemental digital content**

**Table 1:** Number of BPARs, worst severity of BPAR, treatment failure, and chronic nephropathy between the treatment groups (ITT population)

| **Variable** | **Low-dose Tac group  (Group A), (*N*=151)**  *n* (%) | **Standard-dose Tac group  (Group B), (*N*=141)**  *n* (%) |
| --- | --- | --- |
| **Treatment failure*** | 22 (14.6) | 16 (11.3) |
| **At least one BPAR** | 16 (10.6) | 14 (9.9) |
| Banff grade Ia | 6 (4.0) | 6 (4.3) |
| Banff grade Ib | 2 (1.3) | 3 (2.1) |
| Banff grade IIa | 5 (3.3) | 3 (2.1) |
| Banff grade IIb | 2 (1.3) | 2 (1.4) |
| Missing severity | 1 (0.7) | 0 (0.0) |
| **Graft loss** | 6 (4.0) | 2 (1.4) |
| **Death** | 1 (0.7) | 2 (1.4) |
| *BPAR, graft loss, or death | | |
